# Adjuvant endocrine monotherapy for postmenopausal early breast cancer patients with hormone-receptor positive: a systemic review and network meta-analysis

**DOI:** 10.1007/s12282-017-0794-8

**Published:** 2017-07-28

**Authors:** Zhu Yu, Xiaojing Guo, Yicheng Jiang, Lei Teng, Jinwu Luo, Pengfei Wang, Yunsheng Liang, Haitian Zhang

**Affiliations:** Department of Gastrointestinal and Gland Surgery, Sino-Germany Standard Diagnosis and Treatment Center of Breast Surgery, First Affiliated Hospital of Guangxi Medical University, Nanning, Guangxi Zhuang Autonomous Region China

**Keywords:** Endocrine monotherapy, Early breast cancer, Postmenopausal women, Network meta-analysis

## Abstract

**Background:**

In patients with hormone receptor-positive postmenopausal of early stage breast cancer, adjuvant endocrine monotherapies include letrozole, anastrozole, exemestane, toremifene and tamoxifen. But the optimum regimen remains controversial.

**Methods:**

PubMed, Cochrane Database and ClinicalTrials.gov were systematically reviewed of abstract for randomized-controlled trials (RCTs) to assess the efficacy of tamoxifen, letrozole, exemestane, anastrozle and toremifene for postmenopausal patients with hormone-receptor positive (HR+), who have not received prior therapy for early stage breast cancer. The outcomes were measured by disease-free survival (DFS) and overall survival (OS). We evaluated relative hazard ratios (HRs) for death of different therapies by combination hazard ratios for death of included trials. The SUCRA values were used to evaluate the rankings of efficacy for these monotherapies.

**Results:**

A total of fourteen studies including 19,517 patients in our research were absorbed and estimated. The superiority of efficacy for DFS were 5-year letrozole and 10-year tamoxifen (SUCRA values 0.743/0.657) in all comparisons. A more efficient SUCRA values for OS were 5-year Exemestane, 5-year letrozole and 10-year tamoxifen (0.756/0.677/0.669).

**Conclusions:**

Clinically important differences exist between commonly prescribed different adjuvant endocrine monotherapy regimens for both efficacy and acceptability in favor of exemestane and letrozole. 10-year tamoxifen for early breast cancer patients is noninferior to 5-year anastrozle, and might be the best choice where aromatase inhibitors (AIs) are not easy to acquire.

## Introduction

Early breast cancer is a kind of invasive cancer that has not proliferated beyond the breast or the axillary lymph nodes [1]. Worldwide, breast cancer is by far the most frequently cancer occurs to women population. Data from the National Center for Health Statistics (NCHS) showed that breast cancer was the most common cancer diagnosed among US women, accounting for nearly 29% cancers. It had been approximately 40,290 females died of breast cancer in 2011 in America and was second only to lung cancer [2]. The evidence showed that breast cancer was the most common cancer in China in 2011 as well, and the 5-year morbidity was 156/100,000 [3]. The therapeutic strategies for breast cancer mainly include surgery, chemotherapy, endocrine therapy, radiation therapy and targeted therapy. Endocrine therapy remains the first effective systemic treatment for women patients with hormone receptor-positive breast cancer [4].

Tamoxifen, as an antiestrogen drug, has been used in patients with hormone-receptor positive (HR+) breast cancer since 1977. Moreover, it has been proved that adjuvant tamoxifen for 5 years is effective and can reduce the recurrence rate and mortality rate [5]. Simultaneously, ATLAS trial [6] and aTTom trial [7] showed that continuing tamoxifen 10 years or over 10 years has demonstrated carryover benefit for the improving disease-free survival (DFS) and overall survival (OS), compared with used less than or equal to 5 years. Aromatase inhibitors, which include letrozole, exemestane and anastrozle, are applied to clinic for postmenopausal women with oestrogen receptor (ER+) and/or progesterone receptor (PR+) early breast cancer as well [8, 9]. The ATAC trial [10] and BIG 1-98 trial [11] informed that the better efficacy and safety of anastrozole and letrozole monotherapy over tamoxifen for postmenopausal women with ER(+) disease. The comparison of exemestane and anastrozole as 5-year adjuvant monotherapy in MA.27 trial revealed neither to be superior in terms of breast cancer outcomes [12]. Update clinical Face trial [13] in 2016 demonstrated that the equal efficacy in treatment with letrozole and anastrozole for patients.

Above all, different protocols can be selected in the endocrine therapy for early breast cancer, which makes it difficult to choose the empirically superior treatment. No comparison among 10-year tamoxifen and AIs can be found, so far. Hence, the Bayesian network meta-analysis, which combines direct evidence and indirect evidence and compares the efficacy of different monotherapies based on disease-free survival and overall survival, thereby providing an optimum regimen for women with estrogen-positive early breast cancer.

## Methods

### Search strategy and selection criteria

The systematic review was conducted, in terms of PRISMA (Preferred Reporting Items for Systematic Reviews and Meta-Analyses) guidelines [14]. We searched PubMed, Cochrane Database and ClinicalTrials.gov of abstracts for randomized-controlled trials performed using the following search terms: “early breast cancer” and “endocrine therapy” before 31st, December 2016. The adjuvant endocrine therapy includes letrozole, tamoxifen, exemestane, anastrozle, and toremifene. Randomised controlled trials were selected in postmenopausal women with hormone receptor-positive diagnosed early breast cancer. All available studies were searched, including their bibliographies for other relevant publications. If a same study was published in different publications and much same data exited, only the most recent, largest or complete study/data was used in the analysis.

### Data extraction and assessment for risk of bias

Two independent investigators (Zhu Yu, Xiaojing Guo) extracted data and information into an electronic database, which include patient characteristics, inclusion and exclusion criteria, therapy protocols and outcome data (overall survival rate and disease-free survival rate). Analysis was conducted according to recent reports and every study was assessed by the same investigator according to Cochrane risk of bias method [15]. If some important concerns about bias were not appeared in the other domains in the tool, they will be included in other bias.

### Statistical analysis

Not only censoring information was considered, but also time-to-event information can be provided and confounders have been adjusted for HRs [16], which make the reported adjusted HRs were the preferred outcome measure. When HRs were not reported, we generate the HRs from published Kaplan–Meier with the method described by Guyot P [17] and Diaby V [18]. The consistency was assessed by the direct comparison between pooled HRs from the network meta-analysis and corresponding HRs from original results. Network meta-analysis was conducted by WinBUGS 1.4.3 (MRC Biostatistics Unit, Cambrige, UK). The median of the posterior distribution was used as a point estimate for the treatment effect size. After ensuring that posterior distributions were roughly normally distributed, a 95% credible interval (CrI) was derived from the 2.5th and 97.5th percentiles. We assessed model fit using three criteria based on the deviance and node-based residuals. Inconsistency was defined as the difference between the pooled direct and indirect evidence with a 95% CI excluding 1. Three different sets of starting values to fit the model, yielding 150,000 iterations (50,000 per chain) to obtain the posterior distributions of model parameters [19, 20]. The Deviance Information Criteria (DIC) value and residual deviance statistics were applied to Bayesian model selection. The smaller DIC value, the more suitable model [21]. Furthermore, as an alternative ranking method, the surface under the cumulative ranking curve (SUCRA) was calculated to assess the effects. SUCRA values range from 0 to 1, where 1 reflects the best effect with no uncertainty and 0 reflects the worst effect [22].

## Results

**Fig. 1:**
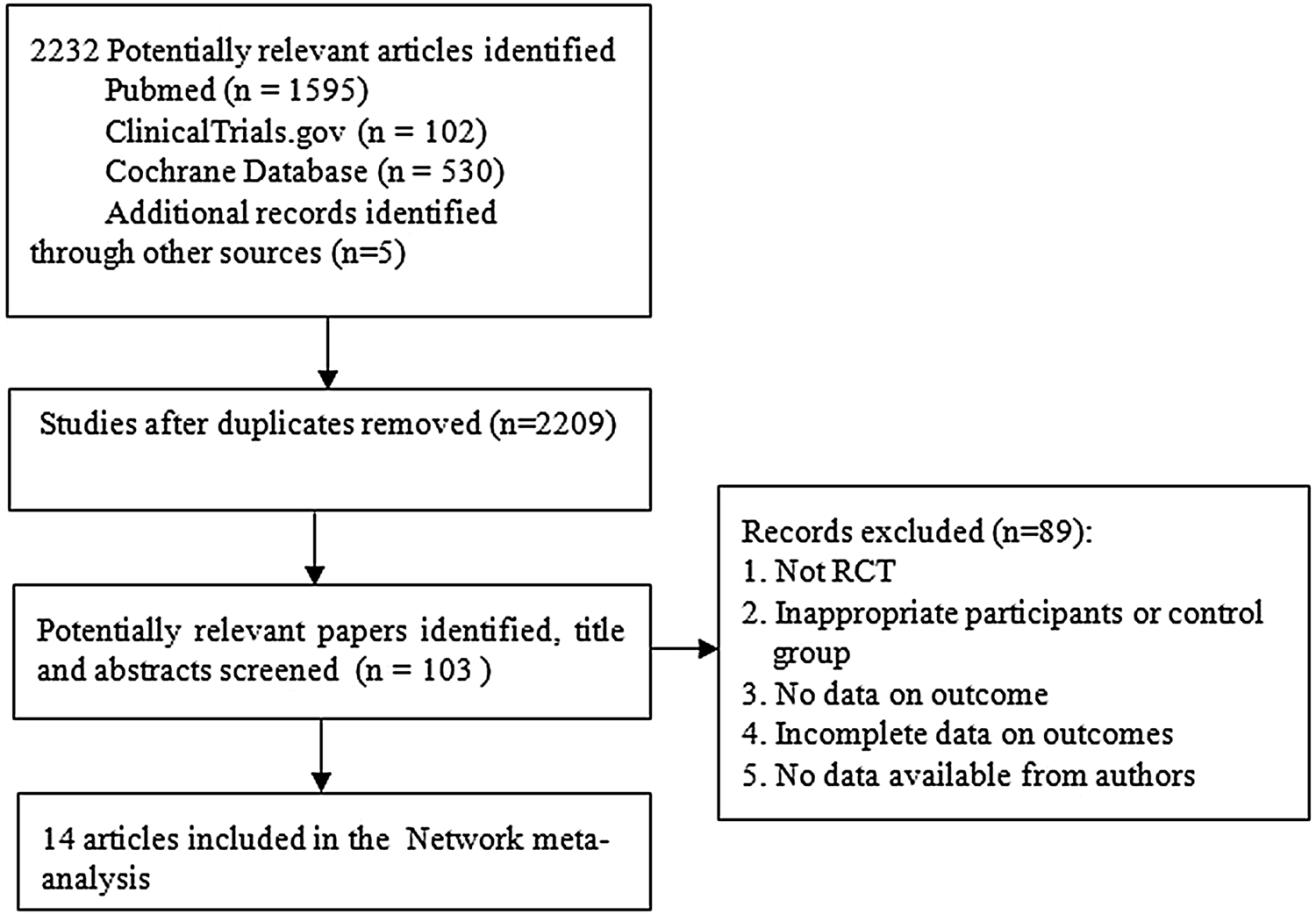
Flow of information through the different phases of the network meta-analysis

We identified 2227 references for reviewing the titles and abstracts from the PubMed (1595), Cochrane Database (530) and ClinicalTrials.gov (102). Finally, by the full text of potentially eligible articles, 14 studies were concluded in the study (Fig. 1). The latest publication of each trial was used for the network meta-analysis, as cited in the main publication.

**Fig. 2:**
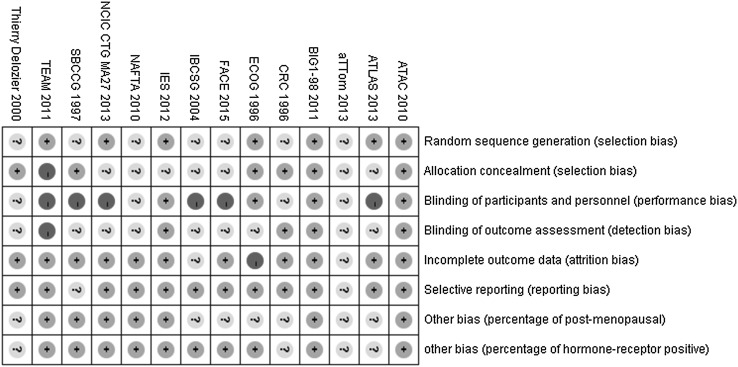
Cochrane risk of bias tool assessment (+: low risk of bias; −: high risk of bias; ?: unclear risk of bias). Other bias: percentage of post-menopausal and HR(+): low risk: ≧50%; high risk of bias: <50%; unclear risk of bias: not mentioned in the article

The characteristics of the 14 studies are summarized in the Table 1. The CRC trial [23], the ECOG trial [24] and the study by Thierry Delozier [25] were retrieval from the bibliographies for other relevant publications. HRs of DFS were reported in 13 studies, which involve the CRC trial, SBCCG trial, IBCSG trial, ATAC trial, NAFTA trial, BIG 1-98 trial, TEAM trial, IES trial, MA.27 trial, ATLAS trial, aTTom trial, Face trial and study by Thierry Delozier [6, 7, 11–13, 23, 25–31], and synthesized in the ECOG trial [24]. OS were reported in only 12 trials and could not be estimated in ECOG trial and study by Thierry Delozier. 12 studies included in the study have been published as manuscripts and most of them have a low risk of bias. The aTTom trial [7] was presented as the consequence of a conference and only an abstract of the Face trial [13] was searched. Hence, it is difficult to judge the bias for the Face trial and the aTTom trial (Fig. 2).

**Table 1:** Summary of randomized-controlled trials of adjuvant endocrine monotherapy for early breast cancer patients

Study	Intervention arm	HR for DFS	HR for OS	Follow-up time	Patients number	Percentage of post-menopausal	Percentage of HR (+) tumor	Median age (years)
CRC (1996)	20 mg daily Tam for 5 years 20 mg daily Tam for 2 years	0.81 (0.69–0.98)	0.89 (0.69–1.15)	NA	1467 1470	NA	NA	51 61
ECOG (1996)	20 mg daily Tam for 10 years 20 mg daily Tam for 5 years	0.448 (0.22–0.91)	NA	5.6 years	73 67	NA	73 (100%) 67 (100%)	NA
SBCCG (1997)	40 mg daily Tam for 5 years 40 mg daily Tam for 2 years	0.80 (0.66–0.96)	0.83 (0.66–1.05)	5.5 years	1096 1114	1096 (100%) 1114 (100%)	1096 (100%) 1114 (100%)	NA
Thierry Delozier (2000)	20–40 mg daily Tam for 10 years 20–40 mg daily Tam for 3 years	0.79 (0.64–0.98)	NA	70 months 30 months	1220 1215	NA	NA	62.8
IBCSG (2004)	60 mg daily Tor for 5 years 20 mg daily Tam for 5 years	0.89 (0.68–1.17)	0.94 (0.64–1.37)	5.5 years	399 374	NA	399 (100%) 374 (100%)	70
ATAC (2010)	1 mg daily A for 5 years 20 mg daily Tam for 5 years	0.86 (0.78–0.95)	0.95 (0.84–1.06)	10 years	2618 2598	2618 (100%) 2598 (100%)	2618 (100%) 2598 (100%)	72
NAFTA (2010)	60 mg daily Tor for 5 years 20 mg daily Tam for 5 years	1.037 (0.72–1.49)	0.951 (0.623–1.451)	59 months	906 907	860 (94.8%) 864 (95.4%)	906 (100%) 907 (100%)	68 67
BIG 1-98 (2011)	2.5 mg daily L for 5 years 20 mg daily Tam for 5 years	0.86 (0.78–0.96)	0.87 (0.77–0.999)	8.1 years	2463 2459	2463 (100%) 2459 (100%)	2463 (100%) 2459 (100%)	61
TEAM (2011)	25 mg daily Tam for 3 years and then 20 mg daily E for 2 years 20 mg daily Tam for 5 years	0.97 (0.88–1.08)	1.00 (0.89–1.14)	5.1 years	4868 4898	4868 (100%) 4898 (100%)	4860 (99.84%) 4888 (99.80%)	64
IES (2012)	20–30 mg daily Tam for 3 years and then 20 mg daily E for 2 years 20–30 mg daily Tam for 5 years	0.81 (0.72–0.91)	0.86 (0.74–0.99)	91 months	2294 2305	2294 (100%) 2305 (100%)	2303 (97.9%) 2314 (97.6%)	NA
MA.27 (2013)	25 mg daily E for 5 years 1 mg daily A for 5 years	1.02 (0.87–1.18)	0.93 (0.77–1.13)	4.1 years	3789 3787	3789 (100%) 3787 (100%)	3766 (99.39%) 3759 (99.26%)	64.2
ATLAS (2013)	20 mg daily Tam for 10 years 20 mg daily Tam for 5 years	0.84 (0.76–0.94)	0.87 (0.78–0.97)	7.6 years	3428 3418	3035 (88.54%) 3044 (89.06%)	3428 (100%) 3418 (100%)	NA
aTTom (2013)	Tam for 10 years Tam for 5 years	0.86 (0.77–0.96)	0.91 (0.80–1.04)	4.2 years	3470 3486	NA	NA	NA
Face trial (2016)	2.5 mg daily L for 5 years 1 mg daily A for 5 years	0.93 (0.80–1.07)	0.98 (0.82–1.17)	65 months	2061 2075	NA	2028 (98.4%) 2053 (98.9%)	62

*Tam* tamoxifen, *Tor* toremifene, *A* anastrozole, *L* letrozole, *E* exemetane

**Fig. 3:**
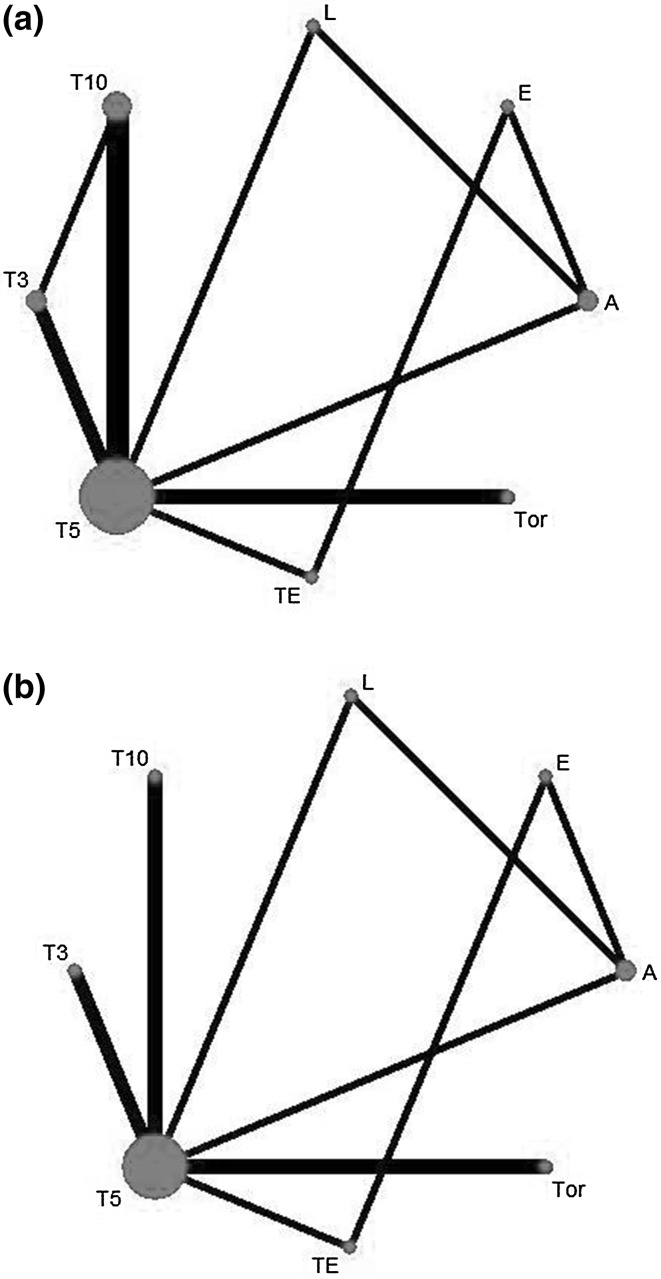
Network of analyzed comparisons. The notes size of DFS (**a**) and OS (**b**) are thickness of the line corresponding to the number of trial per comparison

Figure 3 indicated that the network graph of eligible comparisons. A total of 19,517 patients randomised to receive one of the eight therapy strategies.

It was supposed to use random-effects model for meta-analysis first, in consideration of heterogeneity among studies. It was discovered that there was no significant difference of the Deviance Information Criterion (DIC) between fixed-effected model (DIC = −23.6) and random-effected model (DIC = −21.2). At the same time, the Table 2 presents the results of direct comparisons of univariate meta-analysis and the heterogeneity with *Q* statistics and I2 square in univariate meta-analysis, which indicate that there is no significant difference between these two models. So that the results of fixed-effected network meta-analysis for DFS and OS were presented in Table 3. No significant inconsistency was observed in direct and indirect evidence, by comparing results from traditional pair-wise meta-analysis and network meta-analysis in Table 3.

**Table 2:** The results of direct comparisons and the heterogeneity with I statistics or I2 square of univariate meta-analysis

DFS					OS				
Fixed-effected model HR (95% CI)	Random-effected model HR (95% CI)	Heterogeneity			Fixed-effected model HR (95% CI)	Random-effected model HR (95% CI)	Heterogeneity		
		*Q* value	I2 square	*P* value			*Q* value	I2 square	*P* value
Tor vs T5									
0.940 (0.756, 1.168)	0.940 (0.756, 1.168)	0.437	0.000	0.509	0.945 (0.712, 1.254)	0.945 (0.712, 1.254)	0.002	0.000	0.968
T10 vs T5									
0.884 (0.780, 0.912)	0.837 (0.747, 0.937)	3.176	37.022	0.204	0.886 (0.815, 0.964)	0.886 (0.815, 0.964)	0.267	0.000	0.605
T5 vs T3									
0.812 (0.722, 0.913)	0.812 (0.722, 0.913)	0.404	0.000	0.841	0.857 (0.721, 1.017)	0.857 (0.721, 1.017)	0.157	0.000	0.692

*T3* less than 5 years of tamoxifen, *T5* 5 years of tamoxifen, *T10* 10-year tamoxifen, *Tor* 5-year toremifene

**Table 3:** Pooled hazard ratios for DFS (A) and OS (B) by Bayesian network meta-analysis and pair-wise meta-analysis

A						
T5	0.86 (0.80–0.92) **0.84 (0.79–0.91)**	0.87 (0.80–0.94) *0.86 (0.78–0.95)*	1.20 (1.07–1.34) **1.23 (1.09–1.41)**	0.84 (0.77–0.92) *0.86 (0.78–0.96)*	0.86 (0.76–0.96)	0.95 (0.76–1.17) **0.94 (0.75–1.17)**
	T10	1.02 (0.91–1.13)	1.40 (1.24–1.58) *1.27* (*1.02–1.56*)	0.98 (0.88–1.11)	1.01 (0.88–1.15)	1.11 (0.88–1.39)
		A	1.38 (1.21–1.58)	0.97 (0.88–1.07) *0.93* (*0.80–1.07*)	0.99 (0.88–1.11) *1.02* (*0.87–1.18*)	1.09 (0.86–1.36)
			T3	0.70 (0.61–0.81)	0.72 (0.61–0.84)	0.79 (0.62–1.00)
				L	1.02 (0.89–1.17)	1.13 (0.89–1.41)
					E	1.1 (0.86–1.41)
						Tor

B						
T5	0.94 (0.85–1.03) *0.95* (*0.84–1.06*)	0.89 (0.79–0.99) *0.87* (*0.77–0.999*)	0.86 (0.74–1.00)	0.95 (0.70–1.24) **0.95 (0.71–1.26)**	0.89 (0.81–0.96) **0.886 (0.81–0.96)**	1.17 (0.99–1.39) **1.16 (0.98–1.39)**
	A	0.95 (0.84–1.07) *0.98* (*0.82–1.17*)	0.92 (0.80–1.07) *0.93* (*0.77–1.13*)	1.02 (0.75–1.35)	0.95 (0.84–1.08)	1.26 (1.03–1.53)
		L	0.98 (0.82–1.15)	1.08 (0.78–1.44)	1.00 (0.87–1.15)	1.33 (1.08–1.62)
			E	1.11 (0.79–1.49)	1.03 (0.87–1.21)	1.36 (1.08–1.72)
				Tor	0.95 (0.71–1.27)	1.26 (0.90–1.73)
					T10	1.33 (1.10–1.60)
						T3

The italiced number in one cell is original data from original article. The bold number was the amalgamative HRs which was calculated by pair-wise meta-analysis, if there were two or more articles have HRs of DFS or OS

*CI* confidence interval for traditional meta-analysis, *CrI* credible interval for Bayesian network meta-analysis, *T3* less than 5 years of tamoxifen, *T5* 5 years of tamoxifen, *T10* 10-year tamoxifen, *E* 5-year exemestane, *L* 5-year letrozole, *A* 5-year anastrozole, *Tor* 5-year toremifene, *TE* 2–3 years of tamoxifen followed by 2–3 years of exemestane

**Fig. 4:**
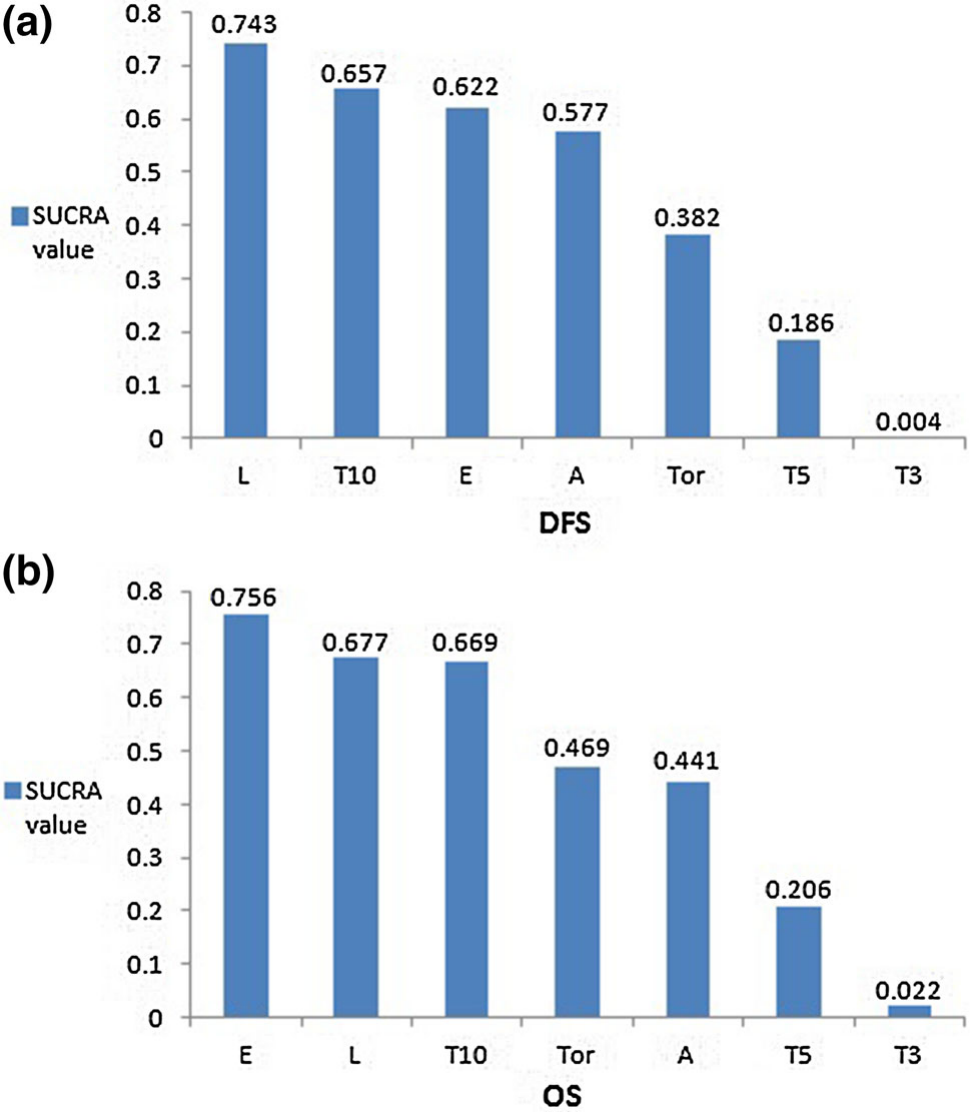
Ranking of interventions with respect to the DFS (**a**) and OS (**b**): SUCRA values

Figure 4 shows the rankings of the eight competing therapy strategies by the SUCRA values based on DFS and OS. For OS, the treatment protocol of exemestane (SUCRA 0.756) ranked in first place for monotherapy, followed by letrozole (SUCRA 0.677), 10-year tamoxifen (SUCRA 0.669), toremifene (SUCRA 0.469), anastrozle (SUCRA 0.441), 5-year tamoxifen (SUCRA 0.206) and less than 5-year tamoxifen (SUCRA 0.022), respectively. Values of SUCRA for DFS showed that letrozole (0.743) had the highest probability of being the best treatment in monotherapy for early breast cancer, which followed by 10-year tamoxifen (SUCRA 0.657), exemestane (SUCRA 0.622), anastrozle (SUCRA 0.577), toremifene (SUCRA 0.382), 5-year tamoxifen (SUCRA 0.186) and less than 5-year tamoxifen (SUCRA 0.004), respectively.

## Discussion

Instead of awaiting only to develop novel hormone therapies, we are instead asking biological questions such as which existing regimen will provide optimal treatment in the clinic.

Upon the study, among the patients who used tamoxifen with different time, it is obvious that the best efficacy was seen for 10-year tamoxifen monotherapy [(DFS: SUCRA 0.657). T10 vs T5 HR: 0.84 (0.79–0.91)]. It is an important result that 10-year tamoxifen can reduce the mortality of early breast cancer [(OS: SUCRA 0.669). T10 vs T5 HR: 0.886 (0.81–0.96)]. These results are in accordance with the results of ATLAS trial, aTTom Trial, CRC trial, SBCCG trial, ECOG trial and study by Thierry Delozier [6, 7, 23–26]. Toremifene, just like tamoxifen, is the one of antiestrogen drugs and binds to estrogen receptors (ERs) [32, 33]. Previous studies indicated that there was no significant difference between tamoxifen and toremifene used with 5 years for patients [30, 31]. In our study, it can be showed that the bigger SUCRA value of toremifene was achieved, either DFS (0.382/0.186) or OS (0.469/0.206), compared indirectly with 5-year tamoxifen. However, there is no head to head study has ever published to compare efficacy of 10-year tamoxifen and 5-year toremifene. Interestingly, a conclusion of the improving DFS (SUCRA 0.657 vs 0.382) and OS (SUCRA 0.669 vs 0.469) by 10-year tamoxifen than 5-year toremifene can be obtained as well, which hinting that it may make more sense to prescribe a 10-year tamoxifen monoherapy to ER-positive patients.

As for DFS, it would be the best choice for patients with 5-year letrozole monotherapy, compared indirectly with 10-year tamoxifen and 5-year exemestane (0.743/0.657/0.622). Meanwhile, the efficacy of 10-year tamoxifen is approximately equivalent to 5-year letrozole [SUCRA value: 0.699 vs 0.677, HR: 1.00 (0.87–1.15)] in prolonging the OS in patients. The SUCRA value of 10-year tamoxifen was greater than 5-year anastrozle for OS [T10 vs A: SUCRA value: 0.669 vs 0.441, HR: 0.95 (0.84–1.08)], which that 10-year tamoxifen for early breast cancer patients is noninferior to 5-year anastrozle. In fact, both DFS and OS in patients with 10-year tamoxifen were prolonged, compared indirectly 5-year anastrozle. To our knowledge, no head-to-head study is currently available to quantify and compare the relative efficacy of 10-year tamoxifen and 5-year anastrozle, and a completely new result emerges out of our study and can provide a credible intervention for early breast cancer in terms of both efficacy and economic benefits.

Aromatase inhibitors, including letrozole, exemestane and anastrozle, are commonly adjuvant endocrine monotherapies applied for early breast cancer. In the Face trial [13], a non-superior efficacy outcome of letrozole was seen versus anastrozle (HR 0.98 [95% CI 0.82–1.17]). In the MA.27 trial [12], the obvious superiority also could not be seen for exemestane (HR 0.93 [95% CI 0.77–1.13]), compared with anastrozle. But our study showed that exemestane and letrozole are more efficacy than anastrozle in terms of OS [SUCRA value: 0.756/0.677/0.441. HR: E vs A: 0.93 (0.77–1.13), L vs A: 0.98 (0.82–1.17)]. Hence, the differences of efficacy among the three aromatase inhibitors can be observed in our study. It is obvious that exemestane is the optimal protocol to improve the overall survival and letrozole is the prefered regimen to improve the DFS.

In a meta analysis, it can be obtained that a reduced recurrence rate approximately 30% and mortality rates about 15% can be maintained by AIs used 5 years than tamoxifen for early breast cancer patients [34]. The similar result was presented for advanced breast cancer. Previous studies presented that the superiority of exemestane, letrozole and anastrozole over tamoxifen for advanced postmenopausal breast cancer females [35–37]. A significant improvement in progression-free survival (PFS) of exemestane was offered, compared with tamoxifen (median PFS: 9.9 vs 5.8 months) [36].It can be seen that a better clinical benefit can be achieved and an improved overall survival by anastrozole when compared with tamoxifen [37]. In view of the above, a significant superior efficacy outcome of aromatase inhibitors compared to tamoxifen for postmenopausal women can be obtained, either early or advanced breast cancer. But the three selective aromatase inhibitors (anastrozole, letrozole, exemestane) have not shown similar anti-tumor efficacy based on our indirect comparison.

A different review for the therapy of tamoxifen was hold by the NSABP-B14 trial and the Scottish trial, which showed that there was no statistically significant difference between 10- and 5-year tamoxifen [38, 39]. All patients with node-negative and ER-positive were randomized to the NSABP-B14 trial, which belong to very early breast cancer stage and few events were seen in those low risk women [40]. Meanwhile, it can be found that the observation time of less than 3 years accounted for more than 50% of the patients. Few patients were randomized to the Scottish trial and inequality in the distribution of the ER-positive patients can be found in the trial. Above all, the NSABP-B14 trial and the Scottish trial were not included in the study.

There are also several limitations in our study. First, not all *P* values pass a specific threshold (0.05) in the 14 analyses, and measurement of disease free survival is less precise than that of overall survival, and might be affected by heterogeneity in follow-up across studies. The ability to provide valid estimates of treatment effect is somewhat limited because trials with different durations of follow-up have been combined. Second, there is no comparison about sequential therapy for early breast cancer in the study. Third, even though strived to get in contact with the key persons in the ECOG trial and study by Thierry Delozier, we could not get the information about overall survival. Furthermore, the reporting of toxic effects was incomplete and inconsistent in the included studies, thus the toxic effects were not conducted in the end. Next, not all of these patients are postmenopausal and suffer from hormone-receptor positive breast tumors in the study, our meta-analysis should be interpreted with some caution, but the results should still provide effective estimates. Finally, a study showed that there was no significant statistical or clinical difference in SUCRA values between different treatments [41], which just provides a numerically favorable treatment difference. Our results simply provide a potential suggestion for the decision made by clinicians and a moderate treatment should be made carefully.

In conclusion, our network meta-analysis suggested that adjuvant endocrine monotherapy with letrozole or exemestane is the optimum endocrine therapy in postmenopausal women with hormone receptor-positive early stage breast cancer. Simultaneously, it is a great possibility that the efficacy of 10-year tamoxifen for early breast cancer patients is noninferior to 5-year letrozole or 5-year exemestane, and even more effective than 5-year anastrozle.
